# Thinking Ability and Creativity Quotient in the landscape of basic education: A contextual and demographic perspective

**DOI:** 10.1371/journal.pone.0350193

**Published:** 2026-05-29

**Authors:** Weeraphol Saengpanya, Ratchaneekorn Upasen

**Affiliations:** 1 Faculty of Education, Thinking, Disposition, and Mental Health-Research Unit Chulalongkorn University, Bangkok, Thailand; 2 Faculty of Nursing, Chulalongkorn University, Bangkok, Thailand; Massachusetts Institute of Technology School of Engineering, UNITED STATES OF AMERICA

## Abstract

Thinking Ability (THI) and Creativity Quotient (CQ) equips students to navigate a complex, fast-changing world. These cognitive functions drive problem-solving, innovation, and adaptability, essential for academic and personal success. In the basic education landscape (elementary and secondary levels), schools worldwide are focusing on promoting core competencies among students. The comparison for THI and CQ based on students’ demographic characteristics is little known. This study examines the levels and demographic variations of THI and CQ among basic education students in Thailand, using a learning-sufficiency framework and key of cognitive and creative developmental theories to explain their development across diverse contexts. A multi-stage random sampling method was employed, involving a total of 1,494 students from schools across various regions. Participants completed a demographic characteristics questionnaire as well as the THI and CQ scales. The results show that the level of THI and CQ among the participants were low to moderate in total and had a statistically significant difference (p < 0.05) when compared based on the demographic characteristics. The study advances theory by providing evidence of distinct patterns across demographic groups. Key implications include adopting stage-appropriate instruction, integrating inquiry- and creativity-based pedagogies, and promoting equity-focused policies to reduce demographic disparities in cognitive and creative development. These findings provide guidance for teachers, educational psychologists, curriculum developers, school leaders, and administrators in strengthening students’ THI and CQ in accordance with their demographic characteristics.

## Introduction

In today’s rapidly evolving world, fostering thinking ability (THI) and creativity quotient (CQ) is essential for preparing students, particularly in basic education (elementary and secondary), to navigate complex challenges and drive innovation. THI includes analytical thinking, critical thinking, problem-solving, logical reasoning, and decision-making, all of which enable learners to process information effectively and develop a deeper understanding of academic content [1]. CQ, on the other hand, refers to an individual’s ability to generate original ideas, think divergently, and apply creative solutions to problems [2]. Students who cultivate both cognitive and creative abilities are better equipped to analyze complex issues, explore unconventional solutions, and approach challenges from multiple perspectives [3]. The creative process reflects a strong connection between cognitive and creative abilities, as it involves the goal-directed use of cognitive resources such as memory, reasoning, and mental flexibility. Creativity emerges when internal attention and purposeful thinking are focused on generating novel and open-ended ideas [4].

Cognitive and creative abilities are deeply intertwined, with cognitive processes forming the foundation for creative performance. Abilities such as memory, attention, problem-solving, reasoning, and executive functioning are essential for processing information, recognizing patterns, and generating ideas [5]. These cognitive functions are critical in creative thinking, which involves applying mental resources in flexible and original ways to produce novel and valuable outcomes [3]. Bloom’s Taxonomy [1] presents a hierarchy of cognitive skills, culminating in “Create” as the highest level—highlighting creativity as an advanced cognitive process. Foundational skills like remembering and understanding support knowledge acquisition, while higher-order skills such as analyzing and evaluating enable the generation of novel, useful ideas [6]. Creativity thus emerges from the integration of these processes. This view aligns with Guilford’s [7] emphasis on divergent thinking and Torrance’s [8] assertion that creativity can be developed through structured cognitive engagement.

Additionally, Research suggests that students who actively engage in both analytical and creative thinking can make meaningful connections between concepts, leading to deeper comprehension and knowledge retention [9]. Additionally, those with a high CQ excel in experimenting, challenging norms, and generating new knowledge, enhancing their academic and professional success [10]. These skills are critical for fostering innovation and problem-solving, which are increasingly recognized as essential competencies in the modern workforce and knowledge-driven economies.

Despite increased scholarly attention to THI and CQ, most existing research continues to prioritize adult populations—such as university students [11], working professionals [12]—leaving basic education settings relatively underexplored. This represents a critical omission, as primary and secondary school years are foundational for shaping cognitive and creative dispositions. Moreover, while certain studies acknowledge demographic variables such as gender and school type [13–14], comprehensive comparative analyses across broader demographic dimensions—such as socioeconomic status, geographic region, and school context—remain scarce [15–16]. The long-term academic implications of CQ, particularly in interdisciplinary learning, are also under-investigated in younger populations [17–18]. At the same time, informal and digital learning environments, including video games and internet use, are emerging as significant factors in the development of thinking skills, yet little is known about how these experiences interact with formal education across demographic groups [19–20]. Institutional and pedagogical constraints—such as curriculum standardization, assessment pressures, and surveillance practices—may further limit the development of creativity in students, with effects likely varying across demographic backgrounds [21].

In Thailand’s basic education system (Prathom 1–6 and Mattayom 1–6), recent reforms promote learner-centered pedagogy and 21st-century skills such as critical thinking, creativity, and digital literacy [22]. However, implementation remains uneven. Classroom practices still often emphasize rote learning, teacher-centered instruction, and standardized testing, limiting opportunities to foster higher-order thinking [23]. Contextual disparities—such as school size, teacher preparedness, and technological access—also impact learning. Students in well-resourced schools’ benefit from enriched curricula and digital tools, while those in smaller or under-resourced settings face limited support for developing thinking and creativity [22,24]. Although alternative models like flipped classrooms and blended problem-based learning show promise [24,25], consistent application is hindered by systemic constraints. Additionally, Gender norms and teacher expectations may shape how boys and girls engage with analytical and creative tasks, leading to varied outcomes in THI and CQ [26].

These disparities underscore the need for research that systematically examines demographic and contextual factors—such as gender, education level, school size, and GPA—to inform inclusive practices and equitable education policy. This study offers a comprehensive view of THI and CQ among Thai basic education students, contributing to educational reform efforts and highlighting their roles across diverse demographic groups. The localized insights also provide a foundation for comparative studies in global educational contexts.

## Literature

### Thinking Ability (THI) and Creativity Quotient (CQ) as foundations tool for learning and innovation in basic education (elementary and secondary education)

#### Thinking Ability (THI) as tool for learning.

THI encompasses a broad range of cognitive skills and serves as a core cognitive tool that enables learners to engage deeply with academic content, adapt to challenges, and solve problems independently through analysis, evaluation, and informed decision-making [27–28]. It includes a range of cognitive abilities of individuals that are expressed when faced with problems in various forms: Analytical thinking for breaking down complex ideas [27] Critical thinking for evaluating credibility and arguments [28], Decision-making and logical reasoning for addressing real-world challenges [29–30], Divergent thinking for creative idea generation [31], and Reflective thinking for fostering metacognition and lifelong learning [32–33]. Beyond a cognitive skill, THI drives knowledge application, innovation, and transfer across educational contexts [9]. This study focuses on three core components of THI—analytical thinking, critical thinking, and decision-making—which play a central role in student development. These abilities help learners process information logically, assess evidence critically, and make sound decisions, forming the foundation for academic achievement and real-world problem-solving.

Analytical thinking refers to the ability to break down complex concepts or situations by examining their components and relationships, recognizing patterns, and identifying key issues [27]. This cognitive process involves diagnosing opportunities, recognizing connections, forming assumptions based on available data, and drawing logical conclusions [34]. Research suggests that students with strong analytical skills perform better in STEM subjects, as they can deconstruct mathematical equations, interpret scientific data, and develop structured problem-solving approaches [9]. A study by Sternberg [5] found that students who engaged in structured analytical exercises demonstrated higher retention rates and deeper conceptual understanding, reinforcing the importance of integrating analytical reasoning into classroom instruction.

Critical thinking involves the objective evaluation of situations and arguments through reasoned reflection, enabling individuals to distinguish fact from opinion, assess information logically, and apply sound judgment in determining appropriate actions [28]. It is essential tool for identified fact from various information important for language learning, humanities, and social sciences, where students must evaluate sources, compare perspectives, and construct well-reasoned arguments. Research conducted by Ennis [35] indicates that students with high critical thinking skills are more independent in their learning, more likely to challenge assumptions, and better at synthesizing diverse viewpoints, which enhances their ability to engage in meaningful discussions and develop well-supported conclusions.

Decision-making is a cognitive process involving the analysis of problems, comparison of alternatives, and selection of the most effective solution based on available information, predicted outcomes, and logical reasoning [29]. In an educational context, this skill is crucial for students as they navigate academic choices, problem-solving scenarios, and social interactions. A longitudinal study by Kuhn [36] found that students who developed strong decision-making skills in school were better prepared for leadership roles, exhibited higher resilience in uncertain situations, and demonstrated stronger problem-solving abilities in professional environments. Recent neurocognitive research highlights emotions as central to decision-making. Emotion-driven values shape both immediate and long-term choices, demonstrating that decision-making integrates emotional and cognitive processes. In education, this supports the use of emotionally rich, real-world scenarios to reflect how learners make authentic decisions.

#### Creativity Quotient (CQ): A tool for innovation development.

CQ measures an individual’s ability to generate new ideas, adapt to novel challenges, and apply divergent thinking in problem-solving [2]. Unlike intelligence quotient (IQ), which focuses on cognitive reasoning and knowledge retention, CQ assesses a person’s ability to think beyond conventional norms, explore alternative solutions, and engage in creative problem-solving [3]. In basic education, CQ is a key driver of innovative learning, enhancing students’ engagement and ability to apply knowledge in diverse ways. Studies show that students with a high CQ exhibit greater curiosity, persistence in problem-solving, and willingness to take intellectual risks [10]. Research conducted by Cropley [6] found that when students were exposed to open-ended problem-solving tasks and project-based learning, their creative potential and critical thinking abilities increased significantly, leading to higher overall academic achievement.

Additionally, Sawyer [37] highlights that schools that incorporate creativity-driven curricula see improvements in student motivation, deeper learning outcomes, and enhanced collaboration skills. CQ is not only valuable for academic learning but also serves as a driving force for innovation development. Research suggests that students who develop creative problem-solving skills early on are more likely to pursue careers in entrepreneurship, research, and technological development [5]. A study by Kim [2] on the relationship between CQ and innovative thinking found that individuals with higher creativity scores were more likely to develop breakthrough ideas, challenge existing norms, and drive advancements in science, technology, and the arts. These findings emphasize the need to integrate creativity-enhancing activities into basic education, such as design thinking exercises, interdisciplinary learning, and hands-on experimentation.

#### Learning sufficiency for Thinking Ability (THI) and creativity development.

The concept of learning sufficiency emphasizes providing learners with meaningful, relevant, and intellectually challenging experiences essential for developing thinking and creativity. It requires moving beyond rote learning toward deep understanding and real-world problem engagement [1]. which posits that educational experiences must engage learners with cognitively rich, meaningful, and developmentally appropriate tasks to foster both THI and creativity [38]. This aligns with broader constructivist and socio-constructivist paradigms that view learning as an active, socially mediated process [39–40].

Additionally, Arievitch et al. [38] offer a compelling integration of Developmental Teaching and Learning (DTL), grounded in cultural-historical activity theory, with Bloom’s Taxonomy of educational objectives. They argue that to ensure learning sufficiency, instruction must progressively guide learners through all six levels of cognitive functioning—from remembering and understanding to applying, analyzing, evaluating, and ultimately creating—and emphasize knowledge as dynamic action that learners must actively perform, not merely memorize.

This developmental pathway supports learners in gradually acquiring and using higher-order thinking skills essential for real-world problem solving and innovation. Such an approach frames learning as both a cognitive and cultural process, where thinking develops not in isolation but through guided participation in meaningful activities. DTL emphasizes that the learner’s growth depends on the cognitive challenge embedded in instructional design and on social interactions that scaffold thinking [38].

Consistent with the DTL model, constructivist theorists like Piaget [39] assert that learners build cognitive structures through active engagement with their environment. Piaget’s view emphasizes logical reasoning, problem-solving, and adaptation, which align with the development of analytical and critical thinking. In parallel, Vygotsky’s [40] socio-constructivist theory highlights the Zone of Proximal Development (ZPD), where learners advance through scaffolded interactions with more capable peers or educators. This process is foundational for both THI and creative exploration.

Learning sufficiency, in this context, refers not only to acquiring knowledge but also to participating in tasks that demand divergent thinking, cognitive flexibility, and decision-making. Craft [41] emphasizes “possibility thinking” as a key driver of creativity in educational settings, where students are encouraged to ask “what if?” and “what might be?”—questions that emerge in environments rich in cognitive challenge and support. Similarly, Runco and Acar [31] argue that creative potential is strongly linked to learners’ capacity for original thinking, a capacity that can be cultivated through carefully structured, yet open-ended, learning opportunities.

The concept of learning sufficiency refers to providing learners with meaningful, contextually relevant, and cognitively challenging experiences to enhance THI and creativity. Learning should progress systematically from understanding to creation through interactive and engaging activities. This aligns with constructivist and socio-constructivist theories, as well as the Developmental Teaching and Learning (DTL) approach, which emphasizes the role of cultural and social contexts in fostering higher-order thinking.

#### Thinking Ability (THI) and Creativity Quotient (CQ) in basic education with demographic perspective.

The study of THI and CQ has grown in prominence in educational research, particularly concerning adult populations such as university students, professionals, and patients with psychological disorders [11–12]. However, there remains a notable gap in research focusing on the developmental trajectory of thinking abilities and CQ within basic education settings (primary and secondary schools). These formative years are critical as they lay the foundation for lifelong cognitive skills, including creative thinking, problem-solving, and decision-making abilities. The demographic perspective, which includes factors such as gender, socioeconomic status, cultural background, and educational environment, is crucial in understanding how different groups of students develop these abilities.

THI, which encompasses various cognitive skills such as critical thinking, problem-solving, and reasoning, plays a pivotal role in students’ academic and life success. Likewise, CQ, often measured through divergent thinking tasks, is vital for innovation, academic achievement, and personal development. Previous studies have shown that creativity and critical thinking are positively linked to academic performance in some contexts [42–43], but the relationships between these cognitive domains are far from uniform. Notably, creativity’s role in education has garnered increasing interest, with studies highlighting its importance in fostering interdisciplinary learning, problem-solving, and adaptability [44]. However, most of this research has been conducted on adult populations (e.g., university students, professionals). The lack of attention to students in basic education leaves a critical gap in understanding how CQ and thinking abilities evolve during these early, formative years. These years are essential for the development of cognitive and creative skills, which are foundational for future academic and professional success.

The role of demographic factors such as gender, socioeconomic status, and cultural background has been shown to influence both thinking abilities and CQ. Gender differences in creativity and critical thinking are well-documented, with studies reporting that females tend to outperform males in critical thinking tasks [26], while males show stronger performance in computational thinking [45]. Additionally, cultural and socioeconomic influences have a significant impact on creativity. For instance, socioeconomic status has been found to have a modest but significant link to creativity, with higher socioeconomic status generally leading to greater opportunities for creativity development [16].

However, research that systematically compares how these demographic factors interact with CQ and thinking abilities in basic education settings remains sparse. Studies focusing on the development of creative thinking across various socioeconomic groups are still limited. Additionally, while studies have noted gender differences in creativity, there is insufficient understanding of how other demographic factors, such as school type, region, and migration background, influence the development of these cognitive skills [46].

Although creativity and thinking abilities have been explored in university and adult populations [11–12], basic education remains underexplored. The demographic variation in thinking and creativity among students from diverse primary and secondary schools needs more empirical investigation. Studies such as those by Cuetos Revuelta et al. [17] and Juriševič and Žerak [18] point to the significance of exploring the long-term development of CQ and thinking skills across different educational stages, but this is still not widely addressed. Understanding the development of creativity across the student life span is critical for early educational interventions.

Studies like those of Shubina et al. [26] highlight the role of informal learning environments (e.g., digital platforms, extracurricular activities) in fostering creativity, but their impact on demographic groups remains underexplored. Informal learning environments, particularly in the digital age, have the potential to offer equitable learning opportunities and support the development of creativity in underserved or marginalized communities. However, more research is needed to explore how these environments interact with socioeconomic and cultural factors to influence creativity and thinking abilities.

While some studies have examined the role of curriculum in shaping creativity [47], there is limited research exploring how national curricula (such as the Merdeka curriculum in Indonesia) shape the development of thinking skills and creative abilities in students from different demographic backgrounds. As Jovanović [48] emphasizes, curriculum design should foster creativity through inclusive pedagogies, yet how curricula interact with demographic factors such as school type and region remain inadequately addressed.

Significant gaps remain in the research on THI and CQ within basic education, particularly concerning demographic differences. While previous studies—mostly focused on adult populations—have shed light on the links between creativity, academic performance, and cognitive development, there is limited understanding of how demographic characteristic variables such as gender, educational stage, school size, and GPA shape these abilities in younger students. Addressing this gap, the present study investigates the levels and variations of THI and CQ among Thai basic education students, with the aim of informing more inclusive and equitable educational practices.

#### Objectives of the study.

This study aims to examine the levels and demographic variations in thinking abilities (THI) and creativity quotient (CQ) among basic education students in Thailand. Anchored in the concept of learning sufficiency and informed by key theories of cognitive and creative development—particularly those of Piaget, Vygotsky, and Guilford—it explores how thinking abilities and CQ emerge and develop in relation to contextual and demographic diversity. By analyzing differences based on gender, educational stage, school size, and cumulative GPA, the study seeks to deepen the understanding of how individual and environmental contexts contribute to variations in THI and CQ during the formative years of education.

### Theoretical framework and hypotheses

This study draws on an integrated framework combining learning sufficiency and key theories of cognitive and creative development—Piaget, Vygotsky, and Guilford—to explore how thinking ability (THI) and creativity quotient (CQ) emerge in basic education, particularly in relation to contextual and demographic diversity.

Learning sufficiency emphasizes meaningful, contextually relevant experiences that foster thinking and creativity rather than rote memorization [1]. The Developmental Teaching and Learning (DTL) model aligns with Bloom’s Taxonomy as a scaffold for cognitive development, guiding learners from remembering to creating to promote higher-order thinking and creative problem-solving [38]. Learning is thus viewed as both a cognitive and sociocultural process.

Jean Piaget’s theory of cognitive development explains how learners progress through stages of intellectual growth, with the concrete and formal operational stages supporting reasoning and problem-solving central to THI [49,50]. Lev Vygotsky’s sociocultural theory complements this by emphasizing social interaction and the Zone of Proximal Development, where scaffolded collaboration with teachers and peers promotes higher-level thinking and creativity [40].

J. P. Guilford’s Structure of Intellect theory highlights divergent thinking—fluency, flexibility, and originality—as a core component of creative functioning underlying CQ [7]. Learning environments that promote exploration and open-ended inquiry enhance creativity and innovation [2,3,6,37]. Within this framework, THI includes analytical thinking, critical thinking, and decision-making, shaped by educational practices and social interaction [27–29].

In the context of Thai education reform emphasizing equity, innovation, and inclusivity, factors such as gender, educational level, school size, and academic performance may influence the development of THI and CQ. Students in well-resourced schools often benefit from enriched curricula and digital tools, whereas those in smaller schools may have fewer opportunities for critical and creative engagement. Overall, THI and CQ reflect interactions among cognitive processes, sociocultural influences, and demographic contexts, underscoring the need for equitable and developmentally appropriate strategies in basic education.

#### Hypothesis of the study.

This descriptive research aimed to investigate and describe thinking ability (THI) and creativity quotient (CQ) among basic education students which categorized by the demographic variables.

This study hypothesizes that students’ levels of THI and CQ differ significantly based on gender, educational stage, school size, and cumulative GPA. As follow:

H1 (Gender): Female students are expected to score higher in critical thinking and decision-making abilities, especially in reflective and value-based reasoning, consistent with findings that girls often outperform boys in verbal and evaluative reasoning tasks [26]. In contrast, male students may show stronger analytical thinking, particularly in structured, logic-based tasks [45], and may perform better in specific aspects of creativity, such as originality.
H2 (Educational Stage): Students in higher educational stages (e.g., lower secondary vs. primary) are hypothesized to exhibit more developed critical thinking, analytical reasoning, decision-making, and CQ, as cognitive abilities mature over time and are influenced by greater exposure to complex academic content [7,39,40].
H3 (School Size): Students from larger schools are expected to achieve higher levels across all domains, due to enhanced access to academic resources, diverse peer interactions, and more varied extracurricular learning opportunities—factors shown to support cognitive and creative growth [46]. These enriched environments may foster both thinking abilities and creativity, particularly when they encourage interdisciplinary and experiential learning [44].
H4 (Cumulative GPA): A higher GPA is anticipated to positively correlate with critical thinking, analytical thinking, decision-making, and CQ, as academic success often reflects the capacity to engage in sustained problem-solving, logical analysis, and innovative thinking [42,43]. GPA may serve as an indicator of students’ engagement in cognitively demanding tasks that cultivate both reasoning and creativity.

**Fig 1:**
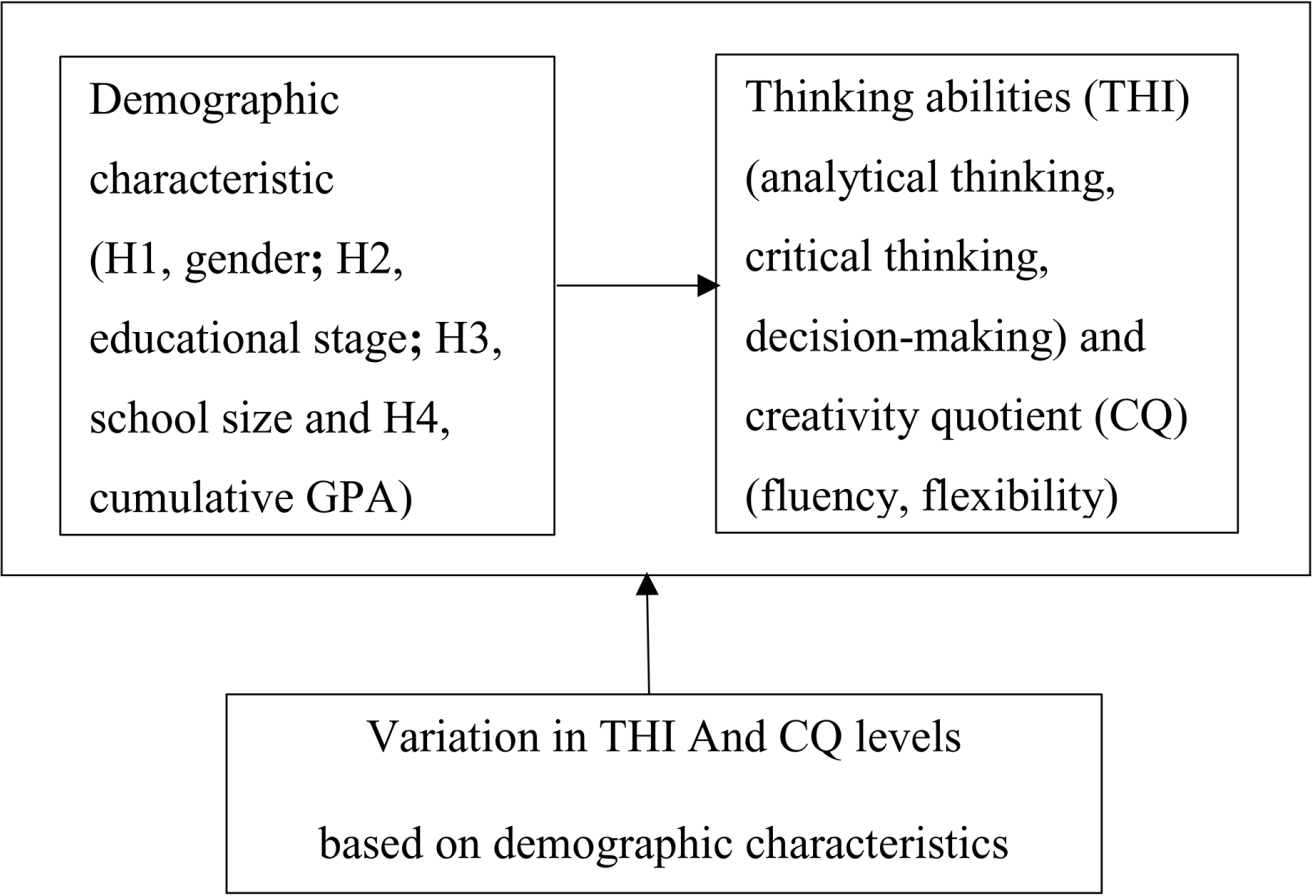
Hypothesis of the study.

These hypotheses aim to address gaps identified in previous research by systematically examining how diverse demographic characteristics are associated with the development of THI and CQ in basic education. The hypotheses for this study are presented in Fig 1.

## Methods

### Study design and setting

This study employed a descriptive–comparative research design with a hypothesis-testing approach to examine how demographic characteristics are associated with thinking ability (THI) and creativity quotient (CQ) among basic education students. Although the sampling procedure was descriptive and conducted at a single time point, the study utilized inferential and hypothesis-testing statistical analyses to examine group differences. It forms part of a larger research project titled *“Thinking and Innovative Behavior of Basic Education Students.”* The aim of the study was to describe variations in THI and CQ across key demographic categories, including gender, educational stage, school size, and GPA. The research was conducted in schools under the jurisdiction of the Office of the Basic Education Commission (OBEC), which operates under the Ministry of Education in Thailand. These settings were selected to ensure the representation of diverse educational contexts within the Thai basic education system.

### Participants

After receiving approval from the research ethics committee, the Second Allied Academic Group in Social Sciences, Humanities and Fine and Applied Arts at Chulalongkorn University (Approval No. 660233), a multi-stage random sampling method was employed to ensure a representative and demographically diverse sample. The study began with the selection of schools from four major regions of Thailand—North, Northeast, Central, and South—to capture regional variation within the basic education system. A total of sixteen basic education schools were randomly selected, with four schools from each region, including two primary schools and two secondary schools. To be eligible for inclusion, schools were required to operate under the Office of the Basic Education Commission (OBEC), offer a complete basic education program, and have students enrolled at either the primary or secondary level. Schools offering only specialized or alternative curricula—such as vocational or special education programs—were excluded.

Within each selected school, classrooms were randomly chosen from eligible grade levels, and all students in those classrooms were invited to participate in this study. To be eligible, student participants were required to be currently enrolled in one of the selected OBEC schools, able to communicate in Thai, and willing to participate in the study. Students who submitted incomplete responses or whose data were deemed invalid were excluded from the final analysis.

In total, 1,494 students participated in the study. Of these, 45.45% (n = 679) were male and 54.55% (n = 815) were female. With regard to educational level, 49.00% (n = 732) were enrolled in primary education and 51.00% (n = 762) in secondary education. Based on educational stage, 23.90% (n = 357) were in Stage 1 (Grades 1–3), 25.10% (n = 375) in Stage 2 (Grades 4–6), 25.50% (n = 381) in Stage 3 (Grades 7–9), and 25.50% (n = 381) in Stage 4 (Grades 10–12). In terms of school size, 48.39% (n = 723) attended small schools, 37.75% (n = 564) attended medium-sized schools, and 13.86% (n = 207) attended large or special-sized schools.

### Consent to participate

Informed consent was obtained from all individuals participants included in the study.

### Instruments

This study utilized six sets of instruments, comprising:

1 General Information Questionnaire – Designed by the researchers to gather demographic details such as gender, education level, school type, and GPA.
2-5 Thinking Ability Test Form B: Stages 1–4 (Grades 1–12) – The Thinking Ability Test, Form B, is a 15-item standardized multiple-choice instrument developed by Kanjanawasee et al. [34] to assess key components of thinking ability, including analytical thinking, critical thinking, and decision-making. The test is available in four stage-specific versions corresponding to educational levels: Stage 1 (Grades 1–3), Stage 2 (Grades 4–6), Stage 3 (Grades 7–9), and Stage 4 (Grades 10–12). Raw scores (0–15) are converted into T-scores for comparative analysis. The KR-20 reliability coefficients range from 0.54 to 0.55, indicating moderate internal consistency [34]. Given the multidomain nature of the construct, internal consistency may be attenuated due to the breadth of content coverage. As the present study emphasizes large-scale group comparisons rather than individual diagnostic decisions, the instrument was considered appropriate for examining and comparing thinking abilities across diverse demographic groups.
6 Creativity Quotient Test – Adapted from Snyder et al. [51], this task-based test measures fluency and flexibility in listing uses for a common object within five minutes. Applicable across Grades 1–12, the creativity quotient was calculated using a logarithmic formula. KR-20 reliability: 0.95.

Although based on Torrance’s framework assessing fluency, flexibility, originality, and elaboration [8], and shown to predict future creative achievement [31], the test has limitations, including cultural bias [52], narrow focus on divergent thinking [53] and decontextualized tasks [54]. However, the version used in this study reduces cultural bias through familiar stimuli and objective scoring, making it appropriate for assessing CQ in basic education [51].

The selection of instruments aligns with the study’s objectives and research questions. The Thinking Ability Test Form B offers standardized, developmentally appropriate measures of cognitive skills across educational stages, supporting demographic comparisons. The Creativity Quotient Test captures divergent thinking central to creativity, reflecting theoretical foundations and student variability. These tools enable a comprehensive analysis of how cognitive and creative abilities relate to demographic factors, contributing insights into learning sufficiency and development within Thai basic education.

### Data collection

After receiving approval from the ethics committee and the selected schools, data collection commenced. The researchers sought permission from school directors before proceeding. Data were collected from basic education schools across various regions of Thailand from September 26, 2023, to February 25, 2024. Written informed consent was obtained from the participants, parents, and legal guardians during the same period. Coordinators then scheduled the sessions at convenient times. Participants independently completed the consent forms, demographic questionnaires, and tests, which were subsequently collected for analysis.

### Data analysis

The data analysis began with a preliminary data screening to ensure accuracy, completeness, and suitability for statistical testing**.** Descriptive statistics, including means, percentages, and standard deviations, were calculated using SPSS version 27. Inferential analyses were subsequently conducted to compare Thinking Ability (THA) and Creativity Quotient (CQ) scores across demographic groups using independent samples t-tests and one-way ANOVA. Prior to conducting these comparative analyses, the assumptions of homogeneity of variance were assessed using Levene’s test. If Levene’s test indicated no violation (p > .05), Scheffé’s post hoc test was applied; however, when the assumption was violated (p ≤ .05), Tamhane’s T2 test was used for unequal variances. To control for the inflation of Type I error due to multiple comparisons, both Bonferroni and Benjamini–Hochberg False Discovery Rate (FDR) corrections were applied in the post hoc analysis.

## Results

### Part 1: The level of Thinking Ability (THI) and Creativity Quotient (CQ) among basic education students

The study assessed students’ Thinking Ability (THI) and Creativity Quotient (CQ) using descriptive statistics and distribution analysis. Overall, THI performance was low (M = 5.68 out of 15, SD = 3.02), with a slightly right-skewed (Sk = 0.43) and moderately platykurtic distribution (Ku = −0.71), indicating that most students performed at low to moderate levels, with relatively few high achievers. Standardized T-scores were used to compare THI across educational stages.

Analysis of the three THI subcomponents—Analytical Thinking (THI-A), Critical Thinking (THI-C), and Decision-Making (THI-D)—provides insight into students’ cognitive profiles. THI-A showed a mean of 1.36 out of 4 (SD = 1.13) with slight right skewness (Sk = 0.42, Ku = 0.59), suggesting difficulty in analyzing and breaking down problems. THI-C had a mean of 1.79 out of 5 (SD = 1.38) with similar skewness (Sk = 0.44, Ku = −0.58), reflecting challenges in evaluation and reasoning. These patterns are consistent with long-standing concerns about Thailand’s reliance on rote learning and limited opportunities for questioning and argumentation in classrooms. Research indicates that explicit instruction in reasoning, evidence evaluation, and dialogic learning can strengthen these skills.

THI-D yielded the highest subcomponent score (M = 2.55 out of 6, SD = 1.73), though the distribution (Sk = 0.41, Ku = −0.71) still indicates overall low performance. This comparatively stronger result suggests that some students draw on intuitive or experiential reasoning, which can serve as a foundation for developing more deliberate decision-making skills through systematic scaffolding and reflective practice.

In addition, the uneven cognitive performance and substantial variability across students point to systemic disparities in instructional quality, resource access, and teacher preparedness. Although national curriculum reforms emphasize analytical and critical thinking, classroom implementation often lags, contributing to limited higher-order thinking skills. These findings underscore the need for targeted strategies to support cognitive development and ensure more equitable learning environments across Thailand’s basic education system.

The Creativity Quotient (CQ), measured through fluency (FLU) and flexibility (FLEX), showed a moderate overall mean (M = 5.92, SD = 3.26). The distribution was positively skewed (Sk = 1.06) and leptokurtic (Ku = 1.71), indicating that most students scored at low levels while a small group achieved substantially higher scores. This pattern reflects uneven creative development, likely influenced by variations in instructional practices and learning opportunities.

Fluency (M = 11.14, SD = 9.55) showed high variability and strong right skewness (Sk = 1.01, Ku = 0.63), suggesting that while some students generated ideas in large quantities, many struggled with divergent thinking. Flexibility was similarly limited (M = 3.20, SD = 1.42), with moderate skewness (Sk = 0.76, Ku = 0.97), indicating challenges in shifting perspectives or producing varied ideas.

These findings reflect concerns that Thai classrooms often emphasize convergent thinking and risk-avoidance, limiting opportunities for experimentation and suppressing creativity. Students may therefore hesitate to explore unconventional ideas, reducing both fluency and flexibility. Creativity develops more strongly in environments that encourage risk-taking, open-ended inquiry, and cross-disciplinary, divergent-thinking activities. Overall, the CQ results underscore the need for intentional pedagogical approaches that better nurture creative potential and provide equitable opportunities for creative growth. The details of these results are presented in Table 1.

**Table 1:** Mean, standard deviation, Skewness and Kurtosis for thinking ability (THI) and creativity quotient (CQ) among basic education students.

Variables	*M*	*SD*	*Sk*	*Ku*
Thinking Ability (0–15 points)	**5.68**	**3.02**	**0.43**	**−0.71**
1. Analytical Thinking (0–4 points)	1.36	1.13	0.42	0.59
2. Critical Thinking (0–5 points)	1.79	1.38	0.44	−0.58
3. Decision-Making (0–6 points)	2.55	1.73	0.41	−0.71
Creativity Quotient	5.92	3.26	1.06	1.71
1. Fluency	11.14	9.55	1.01	0.63
2. Flexibility	3.20	1.42	0.76	0.97

### Part 2: The comparison of students’ Thinking Ability (THI) and Creativity Quotient (CQ) scores by gender, educational stage, school size and GPA

This section presents the results of the comparison of students’ THI and CQ scores based on gender, educational level, and school size. And grade point average (GPA)Statistical analyses were conducted to examine differences among these variables, providing insights into how these factors influence students’ THI and CQ. The results of are as follows

#### Comparison of students’ Thinking Ability (THI) and Creativity Quotient (CQ) by gender.

The analysis revealed significant gender differences in both THI and CQ among basic education students. For THI, females scored significantly higher than males on overall THI (p < .01), as well as on critical thinking (p < .01) and decision-making (p < .05), indicating stronger evaluative reasoning and problem-solving. No significant gender difference was found in analytical thinking**,** suggesting comparable analytical skills. For CQ**,** females also obtained higher overall scores (p < .01), particularly in fluency (p < .05) and flexibility (p < .01), reflecting stronger idea generation and cognitive adaptability.

**Fig 2:**
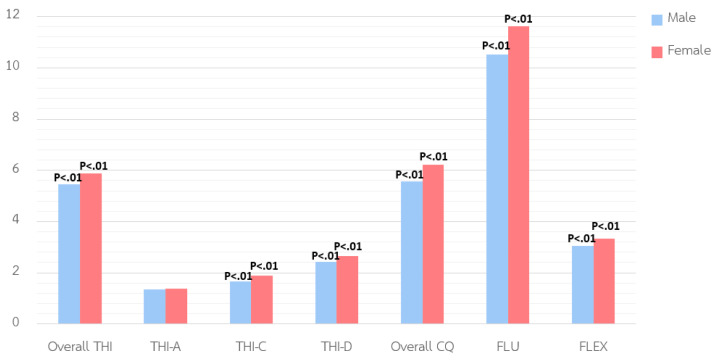
The comparison of THI and CQ by gender.

After correcting for multiple comparisons, differences in critical thinking**,** creativity quotient, and flexibility remained significant under both Bonferroni (.05/7) and False Discovery Rate (FDR) corrections. Overall, THI remained significant only under FDR, while analytical thinking**,** decision-making**,** and fluency did not retain significance. See Table 2 and Fig 2 for details.

**Table 2:** Variance comparison of thinking ability and creativity quotient by gender.

Variable/ Component	Male (n = 679)		Female (n = 815)		Uncorrected p-value/ Bonferroni p-value/ FDR p-value	Summary
	M	SD	M	SD		
Thinking Ability	5.46	3.03	5.87	3.00	.01**/.07/.01**	Female > Male
Analytical Thinking	1.36	1.25	1.37	1.02	.87/1.0/.87	No difference
Critical Thinking	1.67	1.29	1.89	1.44	.00**/.00**/.00**	Female > Male
Decision-Making	2.44	1.75	2.64	1.71	.05*/.35/.07	Female > Male
Creativity Quotient	5.57	3.08	6.21	3.37	.00**/.00**/.00**	Female > Male
Fluency	10.53	9.47	11.63	9.59	.05*/.35/.06	Female > Male
Flexibility	3.04	1.43	3.33	1.41	.00**/.00**/.00**	Female > Male

Note: * p < 0.05**,** ** p < 0.01.

#### Comparison of students’ Thinking Ability (THI) and Creativity Quotient (CQ) by educational stage.

The study reveals significant variations in cognitive abilities across school levels, with younger students (Grades 1–3) consistently outperforming older students across all measured THI domains: overall THI, analytical thinking, critical thinking, and decision-making. All differences were statistically significant at the p < .01 level. This pattern indicates that students in early primary grades demonstrate stronger thinking skills than those in upper primary and secondary levels. The most pronounced differences were observed in critical thinking (THI-C) and decision-making (THI-D), both essential for reasoning and real-world problem-solving. These findings suggest that, rather than steadily developing with age and schooling, certain thinking abilities may decline across the school years, particularly at the secondary level.

In contrast, CQ increased with age, with the highest scores found among high school students (Grades 10–12). The fluency dimension also improved over time, indicating enhanced idea generation with broader learning experiences. However, flexibility peaked in middle school (Grades 7–9) before slightly declining among older students.

**Fig 3:**
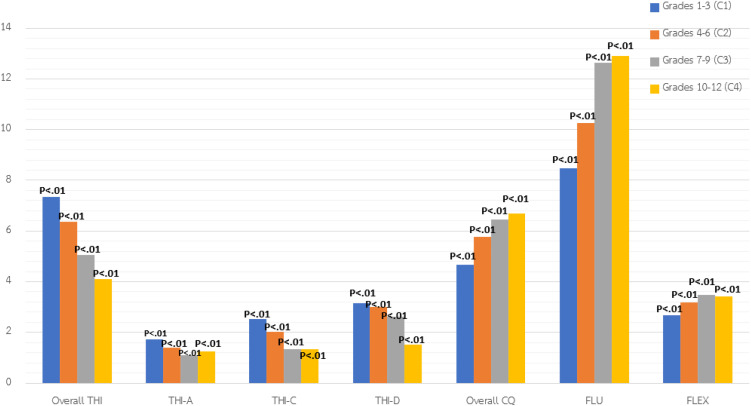
The comparison of THI and CQ by educational stage.

After applying Bonferroni (.05/7) and False Discovery Rate (FDR) corrections, all grade-level differences remained statistically significant across all variables, confirming strong and consistent differences in both THI and CQ across educational levels. See Table 3 and Fig 3 for details.

**Table 3:** Variance comparison of students’ thinking ability and creativity quotient based on their educational stage.

Variable/Component	Level 1: Grades 1–3 (Primary) (n = 375) C1		Level 2: Grades 4–6 (Primary) (n = 375) C2		Level 3: Grades 7–9 (Secondary) (n = 381) C3		Level 4: Grades 10–12 (Secondary) (n = 381) C4		Uncorrected p-value/ Bonferroni p-value/ FDR p-value	Summary
	M	SD	M	SD	M	SD	M	SD		
Thinking Ability	7.34	2.72	6.37	2.98	5.04	2.74	4.11	2.59	.00**/.00** /.00**	C1 > C2, C3, C4; C2 > C3, C4; C3 > C4
Analytical Thinking	1.72	1.04	1.41	1.16	1.09	1.02	1.26	1.20	.00**/.00**/ .00**	C1 > C2, C3, C4; C2 > C3, C4
Critical Thinking	2.53	1.40	2.01	1.22	1.34	1.32	1.33	1.19	.00**/.00**/ .00**	C1 > C2, C3, C4; C2 > C3, C4
Decision-Making	3.14	1.60	2.99	1.92	2.62	1.59	1.51	1.26	.00**/.00**/ .00**	C1 > C2, C3, C4; C2 > C3, C4; C3 > C4
Creativity Quotient	4.66	2.34	5.76	2.98	6.44	3.44	6.69	3.67	.00**/.00**/ .00**	C2, C3, C4 > C1 C3, C4 > C2
Fluency	8.46	8.13	10.25	7.86	12.65	10.56	12.92	10.58	.00**/.00**/ .00**	C3, C4 > C1 C3, C4 > C2
Flexibility	2.67	1.12	3.17	1.37	3.49	1.48	3.43	1.53	.00**/.00**/ .00**	C2, C3, C4 > C1 C3 > C2

Note: * p < 0.05**,** ** p < 0.01.

#### Comparison of students’ Thinking ability (THI) and Creativity quotient (CQ) by school size.

The study revealed significant differences in students’ THI and CQ levels based on school size. Students from medium-sized schools consistently outperformed those from both small and large/extra-large schools in overall THI, analytical thinking (THI-A), critical thinking (THI-C), and decision-making (THI-D). Small school students also outperformed those from large schools in these domains.

**Fig 4:**
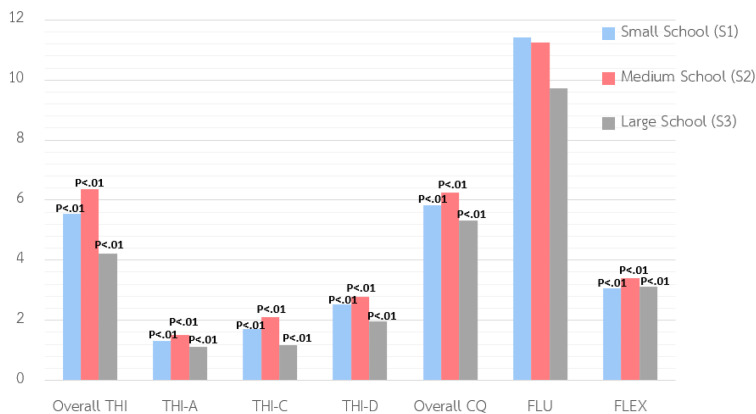
The comparison of THI and CQ by school size.

Similarly, CQ was highest among students in medium-sized schools**,** followed by those in small schools, with large school students scoring lowest. While thinking fluency (FLU) showed no significant differences across school sizes, thinking flexibility (FLEX) was significantly higher among students from medium-sized schools, highlighting their stronger cognitive flexibility. After applying the Bonferroni (.05/7) and False Discovery Rate (FDR) corrections, six variables—THI, THI-A, THI-C, THI-D, CQ, and FLEX—remained significant, indicating consistent school-size effects. FLU did not remain significant. See Table 4 and Fig 4 for details.

**Table 4:** Variance comparison of students’ thinking ability and creativity quotient based on their school size.

Variable/Component	Small sized school (n = 723) S1		Medium-sized school (n = 564) S2		Large/ extra-large sized school (n = 207) (n = 207) *S3*		Uncorrected p-value/ Bonferroni p-value/ FDR p-value	Summary
	M	SD	M	SD	M	SD		
Thinking Ability	5.55	3.05	6.37	2.90	4.23	2.65	.00**/.00**/ .00**	S2 > S1, S3 S1 > S3
Analytical Thinking	1.32	1.11	1.50	1.17	1.11	1.03	.00**/.00**/.00**	S2 > S1, S3 S1 > S3
Critical Thinking	1.72	1.35	2.09	1.34	1.17	1.35	.00**/.00**/.00**	
Decision-Making	2.54	1.81	2.79	1.64	1.96	1.55	.00**/.00**/.00**	
Creativity Quotient	5.83	3.37	6.24	3.10	5.31	3.18	.00**/.00**/.00**	S2 > S1, S3
Fluency	11.42	10.25	11.26	8.22	9.72	10.45	.10/.70/.10	No difference
Flexibility	3.07	1.43	3.39	1.41	3.11	1.38	.00**/.00**/.00**	S2 > S1

Note: * p < 0.05**,** ** p < 0.01.

#### Comparison of students’ Thinking ability (THI) and Creativity quotient (CQ) by GPA.

Students with higher GPAs—particularly those in the 3.51–4.00 range—demonstrated significantly stronger overall THI and analytical thinking (THI-A) (*p* < .01) compared to their lower-GPA peers. In contrast, critical thinking (THI-C) and decision-making (THI-D) did not significantly differ across GPA groups, suggesting these skills may not be fully reflected in academic performance.

Regarding CQ, students with mid-to-high GPAs (3.01–4.00) scored higher on the CQ**,** with top performers achieving the highest scores (*p* < .05). While fluency (idea generation) showed no significant differences, flexibility—the ability to shift perspectives—was significantly higher among high-GPA students (*p* < .05), indicating greater cognitive adaptability. Overall, a higher GPA is associated with stronger analytical thinking and cognitive flexibility, though not necessarily with evaluative or decision-making skills.

After Bonferroni (.05/7) and False Discovery Rate (FDR) corrections, THI-A and FLEX remained significant under both methods**,** while THI and CQ remained significant only under False Discovery Rate (FDR), indicating a weaker effect. Other variables (THI-C, THI-D and FLU) did not retain significance after controlling for multiple comparisons.

**Fig 5:**
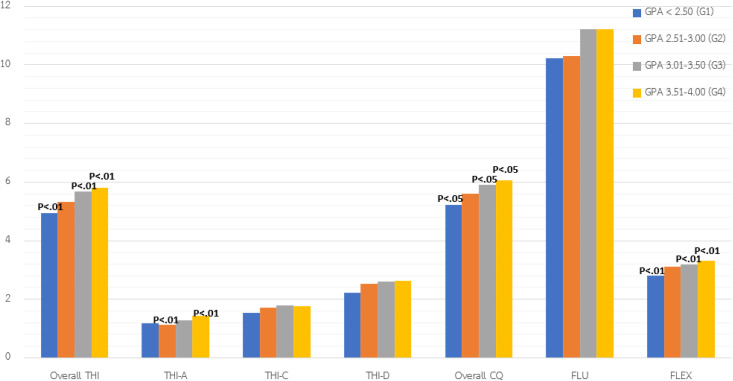
The comparison of THI and CQ by GPA.

See Table 5 and Fig 5 for details.

**Table 5:** Variance comparison of students’ thinking ability and creativity quotient based on their GPA.

Variable/Component	Below 2.50 (n = 131) G1		2.51-3.00 (n = 216) G2		3.01-3.50 (n = 372) G3		3.51-4.00 (n = 606) G4		Uncorrected p-value/ Bonferroni p-value/ FDR p-value	Pairwise Comparison Results
	M	SD	M	SD	M	SD	M	SD		
Thinking Ability	4.93	3.12	5.33	3.05	5.67	3.06	5.81	3.07	.01**/.07/ .02*	G4> G1, G2
Analytical Thinking	1.19	1.07	1.13	.93	1.28	1.04	1.43	1.26	.00**/.00**/ .00**	G4 > G2
Critical Thinking	1.54	1.30	1.71	1.60	1.80	1.26	1.76	1.36	.30/1.00/ .35	No difference
Decision-Making	2.22	1.77	2.52	1.66	2.61	1.85	2.64	1.76	.09/.63/ .12	No difference
Creativity Quotient	5.22	2.97	5.59	2.77	5.90	3.05	6.05	3.35	.03*/.21/ .05*	G3, G4 > G1
Fluency	10.23	9.82	10.29	8.79	11.22	9.37	11.21	9.14	.48/1.00/ .48	No difference
Flexibility	2.81	1.33	3.10	1.32	3.19	1.42	3.32	1.44	.00**/.00**/ .00**	G3, G4 > G1

Note: * p < 0.05, ** p < 0.01.

## Discussions

There are several key points from the findings that warrant discussion.

### The gender differences in Thinking ability (THI) and Creativity quotient (CQ)

The gender analysis revealed that female students significantly outperformed males in overall THI, particularly in critical thinking and decision-making, and also scored higher in CQ (fluency and flexibility). These findings are consistent with those of Musdi et al. [47] and Shubina et al. [26], and may reflect gendered expectations within Thai society and classrooms. Such cultural norms can influence students’ self-perceptions and their engagement in various types of thinking tasks. Moreover, traditional Thai gender roles that emphasize politeness in girls may foster metacognitive sensitivity, which benefits critical and creative thinking. Females’ enhanced verbal reasoning and metacognitive engagement may underlie their higher performance in tasks requiring critical evaluation and decision-making [27,55–58].

This pattern aligns with the hypothesis that female students are expected to score higher in critical thinking and decision-making abilities, especially in reflective and value-based reasoning, consistent with findings that girls often outperform boys in verbal and evaluative reasoning tasks [26]. Conversely, male students may show stronger analytical thinking, particularly in structured, logic-based tasks, and may perform better in specific aspects of creativity, such as originality [45]. However, males may be underrepresented in these areas not due to lower capacity, but because of reduced exposure to activities requiring elaborative or imaginative thinking. This underscores the need for inclusive teaching strategies that challenge these stereotypes and provide equal opportunities to develop a range of thinking skills in all students.

After controlling for multiple comparisons, gender differences were observed mainly in Critical Thinking, Creativity Quotient, and Flexibility**,** with female students showing somewhat higher scores. Thinking Ability showed a smaller effect, remaining significant only under the FDR correction. Differences in Analytical Thinking, Decision-Making, and Fluency were not significant after adjustment, suggesting these differences are less robust. Overall, these results indicate modest gender differences in critical and creative thinking, while general reasoning and decision-making appear more similar between males and females.

### The Thinking (THI) and Creativity Quotient (CQ) differences across educational stages

Comparing educational stages revealed that younger students (Grades 1–3) outperformed older peers in THI, including analytical, critical, and decision-making skills. Piaget’s theory of cognitive development [39] explains this as a reflection of preoperational and concrete operational stages, where children are more exploratory, intuitive, and less risk-averse. Similarly, Vygotsky’s sociocultural theory emphasizes that cognitive development occurs through social interaction and guided learning, suggesting that younger students may benefit from more scaffolded exploration and teacher-supported discovery [39].

This pattern contrasts with theoretical expectations that students at higher grade levels would demonstrate more advanced critical thinking, analytical reasoning, decision-making, and CQ, given that cognitive abilities typically mature with age and exposure to more complex academic content [40–47,49,50]. The observed reversal in THI across grade levels therefore suggests that developmental progression may be shaped not only by age, but also by contextual and instructional conditions [14,60,61].

In the Thai educational context, sustained emphasis on examination performance and extensive content coverage may reduce time for inquiry-based, dialogic, and experiential learning. These structural constraints were further intensified by post-pandemic conditions, including learning loss, unequal digital access, compressed curricula, and reduced classroom interaction. Such disruptions may have disproportionately affected older students, whose curricula require deeper analytical discussion and scaffolded problem-solving.

From the learning-sufficiency perspective, the decline reflects an imbalance in the depth and quality of learning experiences rather than diminished ability. Consistent with Lev Vygotsky’s Zone of Proximal Development (ZPD), higher-order thinking develops through guided dialogue and structured scaffolding. When interactive support and formative feedback are limited, students’ ZPD may not be fully activated, leading to a plateau in cognitive growth despite advancing grade levels. These findings suggest observed stage-related differences; however, given the moderate reliability of the THI instruments, such interpretations should be considered tentative.

Although the finding that younger students outperformed older students in thinking ability appears counterintuitive, cognitive abilities generally become more sophisticated with age and schooling from a developmental perspective. Therefore, the observed pattern is unlikely to reflect a regression in underlying cognitive potential. Rather, it may be associated with contextual and instructional factors. In higher grade levels, increased academic demands, curriculum intensification, and examination-oriented pressures may shift instructional emphasis toward content coverage and test preparation. Such conditions may reduce opportunities for exploratory discussion, open-ended inquiry, and higher-order thinking tasks that foster analytical reasoning and decision-making. Consequently, the differences observed across educational stages may reflect variations in learning environments and assessment practices rather than changes in students’ inherent cognitive capacity.

THI and creativity are closely connected, particularly in early childhood, when intuitive and imaginative reasoning is most pronounced [62]. Piaget’s [39] framework posits a transition from intuitive to logical reasoning as students mature, while Guilford’s divergent thinking model highlights the developmental trajectory of fluency, flexibility, and originality. While logical reasoning is necessary for academic success, this transition may inadvertently constrain divergent thinking and creative expression [63]. Our findings emphasize the need for sustained, developmentally appropriate interventions. This is particularly salient in Thai secondary schools, where standardized testing intensifies and creativity-oriented pedagogy becomes rare. Sustaining students’ THI throughout schooling therefore requires systemic pedagogical shifts, including inquiry-based learning, project-based learning, blended problem-based learning, and flipped classrooms [25,64]. However, the uneven implementation of these strategies, due to structural constraints and uneven teacher preparedness, continues to limit their effectiveness [24].

Interestingly, CQ increased with age**,** peaking in high school. This suggests that exposure to broader experiences and more abstract tasks supports the development of creative fluency [65]. The peak in flexibility during middle school, followed by a decline, may reflect both adolescent openness and later institutional constraints—particularly the pressure of university entrance exams [63]. These patterns align with research showing that young children exhibit more imaginative, intuitive reasoning and are less constrained by logical or evaluative filters [66–67]. Fluency, a key aspect of creativity, also improved with age, indicating that older students generate ideas more effortlessly due to accumulated experience with abstract thinking and complex problem-solving [65]. Overall, these findings suggest that creativity benefits from accumulated experiences, broader knowledge, and diverse learning opportunities [68].

Additionally, after applying Bonferroni (.05/7) and FDR corrections, all grade-level differences remained statistically significant across all variables, confirming the robustness of these developmental trends. This supports the conclusion that thinking ability and creativity consistently evolve across educational stages and underscores the importance of promoting both THI and CQ through developmentally appropriate, scaffolded, and creativity-supportive pedagogical strategies at each level. The integration of Piagetian, Vygotskian, and Guilfordian perspectives, alongside the principles of learning sufficiency, provides a theoretical framework for understanding these patterns and designing interventions that support sustained cognitive and creative development across grades.

### The relevance of school size on Thinking Ability (THI) and Creativity Quotient (CQ)

Findings revealed that students from medium-sized schools outperformed those from small and large schools on both THI and CQ measures. This suggests that medium-sized schools may offer an optimal balance of personalization, peer interaction, and access to resources—conditions conducive to fostering higher-order thinking and creativity [69–70]. From a structural and instructional perspective, this advantage may reflect balanced resource allocation, manageable teacher–student ratios, strong administrative support, and consistent access to learning technologies, which together facilitate student-centered pedagogies that promote analytical thinking, critical thinking, decision-making, and CQ. After applying the Bonferroni (.05/7) and FDR corrections, six variables—thinking ability, analytical thinking, critical thinking, decision-making, creativity quotient, and flexibility—remained significant, indicating consistent school-size effects, while fluency did not remain significant. These results confirm that school size has a meaningful influence on multiple dimensions of thinking and creativity, with medium-sized schools providing a particularly supportive environment.

This pattern contrasts with the hypothesis that students from larger schools are expected to achieve higher levels across all domains, due to enhanced access to academic resources, diverse peer interactions, and more varied extracurricular learning opportunities—factors shown to support cognitive and creative growth [46]. These enriched environments may foster both thinking abilities and creativity, particularly when they encourage interdisciplinary and experiential learning [44]. The observed advantage of medium-sized schools in this study, however, may reflect a more effective integration of personalization and structure—balancing the benefits of resource availability with a supportive social and academic environment. It may be that in very large schools, higher student–teacher ratios and more complex administrative structures are associated with slightly reduced opportunities for individualized academic support, despite greater overall resources. In contrast, the Thai educational landscape presents structural challenges across different school sizes. Small schools frequently face teacher shortages, multi-grade classrooms, and limited access to technology. Additionally, excessive non-instructional duties and administrative burdens reduce teachers’ capacity to focus on instructional quality. Meanwhile, large urban schools, though better resourced, may lack personalized support and student engagement [22]. These structural and instructional disparities may partially explain the performance differences observed across school sizes. These disparities raise concerns about educational equity, particularly in the delivery of 21st-century skills. Uneven access to infrastructure, internet connectivity, and digital literacy continues to hinder efforts to cultivate creativity and thinking skills, especially in under-resourced areas [24]. To address this, educational policies must ensure equitable access to digital tools, teacher training, and innovative pedagogical models across all school sizes and regions.

This finding aligns with prior research highlighting the need to sustain ideational flexibility throughout schooling [71–72]. While innovative learning environments promote the development of these abilities [73], rigid assessments and curriculum constraints can restrict student expression and teacher autonomy [21]. Supportive classrooms that prioritize autonomy, collaboration, and teacher engagement remain essential for fostering both critical and creative thinking [13].

Moreover, broader socio-cultural and regional contexts influence learning outcomes. Regional variations in THI [74] highlight the systemic nature of these disparities. At the same time, differences attributed to school size may also reflect variations in demographic composition, socio-economic background, selective enrollment, or school climate, suggesting that school size may serve as a proxy for broader institutional and contextual factors rather than a direct cause. These findings underscore the importance of developmentally appropriate, context-sensitive, and equity-driven interventions. In particular, the role of school size warrants closer attention, as it significantly shapes the conditions under which THI and creativity are cultivated and sustained.

### Variation in Thinking Ability (THI) and Creativity Quotient (CQ) by GPA

Students with higher GPAs (3.51–4.00) demonstrated significantly greater analytical thinking and flexibility, consistent with research suggesting a link between academic performance and structured reasoning [75]. After correcting for multiple comparisons (Bonferroni and FDR), analytical thinking and flexibility remained significant under both methods. Overall thinking ability and creativity quotient remained significant only under FDR, indicating weaker effects. Critical thinking, decision-making, and fluency were no longer significant. These results indicate that certain facets of thinking and creativity are more closely aligned with GPA, whereas other may develop independently of traditional academic achievement. The study supported the hypothesis that students with higher GPAs would exhibit stronger analytical thinking as academic success often reflects the ability to engage in sustained problem-solving, logical analysis. [42,43].

However, no significant differences were found in critical thinking or decision-making across GPA groups, supporting the argument that such skills rely more on reflective, experiential learning than on traditional academic measures [5,76]. The weak or inconsistent relationship between CQ and GPA further reinforces creativity as a distinct domain [42,77].

The finding that idea fluency did not vary by GPA supports the view that creativity and academic performance are only partially linked. This aligns with studies showing weak or context-specific correlations between creativity and GPA [77–78], and reinforces that creativity is a distinct cognitive domain [42].

Notably, flexibility—a core component of creativity—was consistently higher among top academic performers, reflecting Kapoor et al. [75], who found it predictive of standardized test scores. This suggests that certain facets of creativity, such as adaptability in idea generation, may align with academic achievement, even if more intuitive or original forms of creativity are less tied to grades. Additionally, creativity development depends on more than academic context; supportive classroom climates [43] and arts-based programs [44] also play key roles. This underscores the importance of alternative assessments and learning experiences for developing thinking and creativity. In Thailand, where academic success is still largely measured through test-based evaluations, creativity may not be directly rewarded or nurtured through the mainstream grading system.

## Conclusions

This study provides a comprehensive and contextually grounded understanding of Thai students’ thinking ability (THI) and creativity quotient (CQ) across demographic and educational factors in basic education, framed through learning sufficiency and cognitive–creative development theories. The findings indicate moderately low THI and moderate CQ, with notable differences across gender, educational stage, school size, and GPA. Female students consistently outperformed males, underscoring the role of sociocultural influences, while younger students exhibited stronger thinking abilities and older students demonstrated greater creative fluency. Medium-sized schools appeared particularly conducive to fostering both THI and CQ, suggesting that balanced resources and engagement optimize cognitive and creative growth. Importantly, the weak link between GPA and higher-order thinking skills suggests that conventional academic metrics alone are insufficient to fully reflect or develop these competencies.

The study makes three key contributions. First, it advances theory by elucidating how contextual and demographic factors interact with cognitive and creative development, offering a more integrated and culturally grounded understanding of THI and CQ in basic education. Second, it informs policy by emphasizing the need for equitable cognitive development across educational stages, particularly through resource distribution, stage-appropriate support, and assessment systems that extend beyond GPA-based evaluation. Third, it provides practical implications for instructional design and teacher professional development, advocating developmentally aligned, scaffolded, and socially mediated learning approaches that intentionally cultivate both thinking and creativity.

Overall, the study contributes analytically and practically by identifying structural and contextual conditions that shape students’ cognitive–creative development and by offering actionable directions for strengthening educational reform in Thailand.

### Implementation

To address the moderately low Thinking Ability (THI), moderate Creativity Quotient (CQ), and disparities across gender, educational stage, school size, and GPA, actionable, developmentally aligned strategies are required at instructional, curricular, policy, and institutional levels.

1 Instructional Practices: Enhancing Cognitive Engagement and Creativity

Educators should shift from lecture-based teaching to active, student-centered approaches such as Project-Based Learning (PBL), Inquiry-Based Learning (IBL), and flipped classrooms to strengthen problem-solving, reasoning, and ideational fluency [79–83]. PBL builds collaboration and innovation [79–80]; IBL enhances reasoning and critical thinking [81]; and flipped learning increases participation and cognitive engagement [82–83]. These approaches align with learning sufficiency by supporting student autonomy and foundational mastery [38]. Instruction must be differentiated based on developmental stages: scaffolded, cognitively structured tasks for younger learners [84], and open-ended, autonomy-driven tasks for older students to stimulate ideational flexibility and fluency [59]. The Developmental Teaching and Learning (DTL) model reinforces this differentiation by gradually transitioning from concrete, teacher-led tasks to abstract, self-directed inquiry to foster both cognitive resilience and creativity [38,40,85].

In practice, teachers should operationalize these principles through stage-appropriate strategies. In early primary levels, inquiry-based and exploratory activities supported by guided questioning and structured scaffolding can help sustain curiosity and foundational thinking skills. In upper primary levels, analytical thinking, critical thinking, and decision-making tasks should be intentionally embedded within subject instruction, supported by collaborative problem-solving and formative feedback. At the secondary level, interdisciplinary projects, authentic problem-based learning, and real-world applications should be emphasized to deepen higher-order thinking, autonomy, and creativity. Across all stages, the consistent use of formative assessment, reflective dialogue, and autonomy-supportive classroom practices is essential to maintain cognitive engagement and promote sustained creative development.

2 Assessment Reform: Capturing THI and CQ More Accurately

The weak alignment between GPA and higher-order thinking indicates that traditional grading does not accurately reflect THI or CQ. Educators should integrate authentic assessments such as portfolios, collaborative projects, and performance-based tasks to better measure critical thinking, decision-making, and creative problem-solving [86]. These methods align with learning sufficiency principles and lifelong learning, while also requiring professional development in cognitive scaffolding and culturally responsive pedagogy [87].

3 Curriculum Design: Integrating Cognitive Rigor and Creative Expression

Curriculum developers should embed structured reasoning, argumentation, and conceptual inquiry to build THI [55], alongside open-ended explorations that encourage creativity, ideation, and divergent thinking [31]. In mathematics, include non-routine problems with multiple solution paths; in language arts, use divergent writing prompts and reinterpretation of narratives. Content should be sequenced using cognitive scaffolding aligned with the DTL model to support progressive cognitive development, retention, and transfer [38,40,85,88]. Alignment between curriculum and assessment is critical to avoid reinforcing rote learning. Instructional materials and digital platforms should incorporate creativity challenges, thinking labs, and interdisciplinary projects to promote collaboration and adaptability [89].

4 Policy and Institutional Support: Addressing Disparities and Enabling Innovation

Policy actions should focus on reducing gender-based disparities by promoting inclusive, gender-sensitive pedagogy and challenging cognitive stereotypes through teacher training and curriculum design [90–92]. School size differences indicate that medium-sized schools offer balanced cognitive support; policymakers should therefore promote peer-learning networks where medium-sized schools’ mentor smaller and larger institutions. Support for rural and small schools should include investment in infrastructure, digital access, and teacher incentives [93], while large urban schools benefit from decentralized management and personalized learning strategies [94]. National assessments should expand to include creative problem-solving, reflective writing, and performance tasks, and teacher certification should reward innovative pedagogy aligned with learning sufficiency, cognitive development, and DTL [89]. Digital platforms for micro-credentials, peer coaching, and professional learning can foster continuous improvement, especially in under-resourced contexts [95].

5 Institutional Leadership and School Administrator

School leaders should cultivate environments that promote cognitive engagement, creativity, and reflective learning. This includes establishing innovation labs, makerspaces, quiet reflection zones, and interdisciplinary project areas, while reducing bureaucratic constraints to empower teachers’ autonomy. Leadership should foster partnerships with community organizations, industries, and universities to provide real-world learning experiences and sustain relevance.

For school administrators, it is essential to ensure balanced resource allocation, manageable teacher–student ratios, and equitable access to digital tools across diverse school contexts in order to reduce disparities in opportunities for cognitive development. Equity-focused planning should be prioritized so that all learners experience enriched and supportive learning environments. Administrators should also systematically promote professional development centered on student-centered, inquiry-based, and creativity-enhancing pedagogies, while strengthening teacher learning communities and reflective practice.

Reducing excessive administrative burdens is equally important to preserve teachers’ capacity to deliver cognitively engaging instruction. Streamlined processes allow teachers to focus more effectively on fostering higher-order thinking and meaningful student interaction. Finally, administrators should cultivate a supportive and psychologically safe school climate that values questioning, dialogue, exploration, and intellectual risk-taking, thereby sustaining analytical thinking, critical thinking, decision-making, and creativity across educational stages.

System-wide improvement in THI and CQ requires alignment across instruction, curriculum, policy, and school leadership. Developmentally appropriate, equity-focused, and innovation-driven strategies can foster reflective, creative, and sufficient learning for Thai students.

### Limitations and recommendations for future research

This study provides valuable insights into the interplay between thinking ability (THI), creativity quotient (CQ), and demographic characteristics within the basic education context. Nevertheless, several limitations should be acknowledged to guide future research. First, the cross-sectional design limits causal interpretations and does not capture the developmental progression of THI and CQ. Second, despite the use of multi-stage random sampling, the focus on Thai basic education students restricts the applicability of findings to other educational and cultural contexts. Third, data collection during final examination periods at some schools may have affected student performance due to stress and time constraints. Fourth, while standardized instruments were employed, they may not fully represent the multidimensional nature of THI and CQ, in particular, the THI instrument demonstrated low internal consistency (KR-20 = 0.54–0.55), the moderate reliability may attenuate effect sizes and inflate measurement error and limit the precision of THI-related findings. Therefore, findings based on the THI should be interpreted with caution. Finally, the rapid evolution of educational technology calls for periodic re-examination of the findings to ensure ongoing relevance.

To advance this line of inquiry, future studies should employ longitudinal designs to examine how THI and CQ develop over time and identify key instructional and environmental factors shaping these trajectories. Research should also investigate classroom-level variables—such as teacher beliefs, instructional strategies, and regional disparities—as well as structural challenges like teacher workload in small schools and reduced student engagement in larger institutions. Furthermore, future research should apply multilevel modelling techniques to partition within- and between-school variance and examine contextual predictors. Experimental or quasi-experimental approaches are recommended to assess the impact of targeted pedagogical interventions, including inquiry-based learning, debate, and design thinking, on enhancing THI and CQ outcomes.

Future research should also undertake cross-cultural comparative studies, particularly within ASEAN and East Asian contexts, to explore how cultural norms, assessment practices, and educational policies influence cognitive development. Additionally, studies should consider the role of non-formal learning environments and emerging digital innovations—such as AI-powered platforms, adaptive learning systems, and virtual simulations—in promoting THI and CQ, especially in resource-constrained or rural settings. Furthermore, future research should refine the THI or use higher-reliability measures to reduce measurement error, especially since data collected during exams may affect student performance. These directions will support the development of more inclusive, context-responsive, and evidence-based policies that foster holistic and sustainable student development.
